# Pharmacotherapeutic follow-up in a respiratory intensive care unit: description and analysis of results

**DOI:** 10.1590/S1679-45082018AO4112

**Published:** 2018-06-15

**Authors:** Ana Carolina de Souza e Silva, Domingos Sávio de Carvalho Sousa, Eunice Bobô de Carvalho Perraud, Fátima Rosane de Almeida Oliveira, Bruna Cristina Cardoso Martins

**Affiliations:** 1 Hospital de Messejana Dr. Carlos Alberto Studart Gomes, Fortaleza, CE, Brazil.; 2 Universidade Federal do Ceará, Fortaleza, CE, Brazil.

**Keywords:** Critical care, Pharmacists, Pharmaceutical services, Pharmacy service, hospital, Drug prescriptions, Cuidados críticos, Farmacêuticos, Assistência farmacêutica, Serviço de farmácia hospitalar, Prescrições de medicamentos

## Abstract

**Objective:**

To describe and evaluate the pharmacotherapeutic follow-up by a clinical pharmacist in an intensive care unit.

**Methods:**

A descriptive and cross-sectional study carried out from August to October 2016. The data were collected through a form, and pharmacotherapeutic follow-up conducted by a clinical pharmacist at the respiratory intensive care unit of a tertiary hospital. The problems recorded in the prescriptions were quantified, classified and evaluated according to severity; the recommendations made by the pharmacist were analyzed considering the impact on pharmacotherapy. The medications involved in the problems were classified according to the Anatomical Therapeutic Chemical Classification System.

**Results:**

Forty-six patients were followed up and 192 pharmacotherapy-related problems were registered. The most prevalent problems were missing information on the prescription (33.16%), and those with minor severity (37.5%). Of the recommendations made to optimize pharmacotherapy, 92.7% were accepted, particularly those on inclusion of infusion time (16.67%), and dose appropriateness (13.02%), with greater impact on toxicity (53.6%). Antimicrobials, in general, for systemic use were drug class most often related to problems in pharmacotherapy (53%).

**Conclusion:**

Pharmacotherapeutic follow-up conducted by a pharmacist in a respiratory intensive care unit was able to detect problems in drug therapy and to make clinically relevant recommendations.

## RESUMO

**Objetivo:**

Descrever e avaliar o acompanhamento farmacoterapêutico do farmacêutico clínico em uma unidade de terapia intensiva.

**Métodos:**

Trata-se de um estudo descritivo, com desenho transversal, realizado no per**í**odo de agosto a outubro de 2016. Os dados foram coletados por meio de um formulário de registro, com acompanhamento farmacoterapêutico realizado pelo farmacêutico clínico na unidade de terapia intensiva respiratória de um hospital terciário. Os problemas registrados nas prescrições foram quantificados e classificados, sendo avaliados quanto à gravidade; as recomendações realizadas pelo farmacêutico clínico foram analisadas em relação ao impacto na farmacoterapia. Os medicamentos envolvidos nos problemas foram categorizados utilizando o *Anatomical Therapeutic Chemical Classification System*.

**Resultados:**

Foram acompanhados 46 pacientes, tendo sido registrados 192 problemas relacionados à farmacoterapia. Os problemas prevalentes foram informação ausente na prescrição (33,16%) e com gravidade menor (37,5%). Das recomendações realizadas para a otimização da farmacoterapia, 92,7% foram aceitas, sendo prevalentes aquelas referentes a inclusão do tempo de infusão (16,67%) e a adequação da dose (13,02%), com maior impacto na toxicidade (53,6%). Os anti-infecciosos gerais para uso sistêmico constituíram classe de medicamentos mais frequente nos problemas relacionados à farmacoterapia (53%).

**Conclusão:**

O acompanhamento farmacoterapêutico realizado pelo farmacêutico em uma unidade de terapia intensiva respiratória mostrou-se capaz de detectar problemas na farmacoterapia dos pacientes e realizar recomendações clinicamente relevantes.

## INTRODUCTION

In the hospital setting, the intensive care unit (ICU) is the place where most medication errors occur. This may be related to the fact the inpatients are more likely to have problems considering the critical nature of their diseases, and/or to polypharmacy, use of high-risk drugs and frequent changes in prescriptions.^(^ ^1^ ^-^ ^3^ ^)^ Therefore errors and their consequences are more severe in intensive care patients, since 19% of ICU medication errors are life-threatening, and 42% are clinically relevant to the extent that additional life support measures are required.^(^ ^4^ ^,^ ^5^ ^)^

The term “medication error” is defined as an avoidable event that may lead to inappropriate use of the medication, causing or not harm to the patient. These errors may occur at any stage of drug therapy and include errors in prescription, transcription, dispensing, preparation and administration.^(^ ^6^ ^-^ ^8^ ^)^

In healthcare organizations, implementing systems to detect and prevent prescription errors should be one of the objectives of pharmacovigilance and of the Clinical Pharmacy Service. Hence, a continuous evaluation could be established, with t he purpose of reducing the incidence of errors and – particularly - contributing to identification and report of new events, which are mistakenly considered adverse reactions.^(^ ^9^ ^)^

Pharmaceutical recommendations, defined as a “planned, documented and conducted actions involving healthcare users and professionals, aiming to solve or prevent problems that interfere or may interfere in drug therapy, being an integral part of the pharmacotherapeutic monitoring/follow-up process”,^(^ ^10^ ^)^ are activities carried out by clinical pharmacists. The participation of these professionals in the ICU is one of the strategies that can be adopted to avoid medication errors, since pharmacists provide important information for the safe use of medications.^(^ ^3^ ^)^

The Pharmacy Department of the *Associação de Medicina Intensiva Brasileira* [Brazilian Intensive Care Medicine Association] was established in 2008, emphasizing the importance of the participation of this professional in the intensive care team. In 2010, *Agência Nacional de Vigilância Sanitária* (ANVISA) [National Heath Surveillance Agency] issued the Collegiate Board Resolution Number 7, providing about the general care conditions in ICU, to ensure the presence of a clinical pharmacist at the bedside.^(^ ^11^ ^)^

Pharmacists may participate in many different activities, such as follow-up and monitoring of the medical prescriptions regarding medications, dose, interval, route, dilution, and administration, drug incompatibility; individual assessment of risks; search for updated scientific literature to identify drug administration standards and prepare protocols; participation in promotion of continuing education, fostering knowledge sharing in the multiprofessional team, and providing appropriate technical support; conduction of training sessions; monitoring of adverse events and drug interactions; treatment optimization to reduce hospital costs and thus ensure safe prescription, use and administration of drugs.^(^ ^12^ ^-^ ^14^ ^)^

In healthcare systems, pharmacists are one of the last resources to identify, correct or reduce potential risks associated with therapy. The pharmaceutical recommendations for the rational use of drugs are relevant and acknowledged, but there are still scarce reports on this activity, primarily in special groups of patients.^(^ ^9^ ^)^

There are currently no studies on the role of clinical pharmacists in intensive care for specific risk groups, such as patients with respiratory problems. This fact demonstrates the need for research that contributes to professional development, promotion of rational use of drugs, by critical analysis of risks and benefits of the therapies proposed, and analysis of drug prescriptions prior to dispensing and administration to patients.

## OBJECTIVE

To describe the pharmacotherapeutic follow-up by a clinical pharmacist at a respiratory intensive care unit.

## METHODS

A descriptive cross-sectional study conducted at the respiratory ICU of *Hospital de Messejana Dr. Carlos Alberto Studart Gomes* (HM), in Fortaleza, (CE, Brazil) Brazil, from August to October 2016. The ICU had eight beds for acute and chronic patients who required intensive life support. Patients presented varied clinical conditions, including exacerbated chronic obstructive pulmonary disease, pneumonia, tuberculosis, and other respiratory problems.

The convenience sample included all pharmacotherapeutic follow-ups of patients admitted to the ICU from August to October 2016, and conducted by a clinical pharmacist. Patients with incomplete follow-up form that hindered data analysis, and/or those admitted to the ICU for less than 24 hours were excluded.

During the pharmacotherapeutic follow-up, the results of laboratory tests and records by the multiprofessional team in the medical charts, including medical prescriptions, were analyzed.

The problems and pharmaceutical recommendations documented in the follow-up forms were quantified and classified according to an adapted version made by Costa and Martins.^(^ ^8^ ^,^ ^15^ ^)^ The adjustment was made to include the pharmacotherapy-related problems considered frequent in adult ICU specialized in respiratory conditions.

The severity of problems related to drug therapy was analyzed by a method adapted from Overhage et al., and modified by Fernandez-Llamazares et al. and Costa.^(^ ^8^ ^,^ ^16^ ^,^ ^17^ ^)^ The classification of Farré Riba et et al.,^(^ ^18^ ^)^ was used to analyze the impact of pharmaceutical recommendations, considering as “impact on efficacy” the recommendations that enabled better use of medication by patients to achieve the planned therapeutic goals, and including the recommendations that improve care delivered. The recommendations classified as “impact on toxicity” were those that enabled reducing risks when patients used a drug.

The medications involved in the pharmacotherapy-related problems were classified according to the Anatomical Therapeutic Chemical (ATC) classification system.^(^ ^19^ ^)^

Data analysis was conducted using Excel software for tabulation and cross-referencing of variables using the Epi-Info program v.3.5.1. Numerical variables were described as means and standard deviations, and categorical variables as proportions.

The study was designed according to guidelines and regulatory norms of research involving human beings (CNS: 466/2012). It was submitted to the Internal Review Board of HM, and approved under number 1.536.402, CAAE: 55297316.6.0000.5039. Data collected were treated as confidential, with no identification of patients.

## RESULTS

A total of 46 patients were followed up during the study period. The most frequent diagnoses were sepsis/septic shock (17.34%), chronic obstructive pulmonary disease (15.30%) and pneumonia (11.22%), with a mean of 2.1 diagnoses per patient (standard deviation (SD)±1.0; minimum: one diagnosis; maximum: four diagnoses). The mean length of stay at respiratory ICU during the study period was 14.7 days (SD±12.2; minimum: 1 day; maximum: 60 days), and 63% of patients were transferred to the ward. There were more males (63%). Most patients were aged 66-80 years (34.8%) and >80 years (21.7%), with mean age 66.5 years (SD±16.1; minimum: 25 years; maximum: 91 years) (Table 1).

**Table 1:** Characteristics of the study population at a respiratory intensive care unit

Variables		n (%)
Sex		
	Male	29 (63.0)
	Female	17 (37.0)
Age group, years		
	25-35	1 (2.2)
	36-45	4 (8.7)
	46-55	6 (13.0)
	56-65	9 (19.6)
	66-80	16 (34.8)
	>80	10 (21.7)
Discharge from ICU		
	Ward	29 (63.0)
	Death	17 (37.0)
Length of stay at ICU, days		
	<10	22 (48.0)
	11-20	17 (37.0)
	21-30	4 (8.7)
	31-40	1 (2.2)
	> 41	2 (4.4)

ICU: intensive care unit.

We analyzed 192 problems related to drug therapy registered in the pharmacotherapy follow-up for 528 prescriptions analyzed. The most prevalent were missing information on prescription (33.16%), doses higher than appropriate (12.43%), and unavailability (shortage) (9.84%) (Table 2). Regarding the problems identified, the clinical pharmacist registered 192 recommendations made to the multiprofessional team, with acceptance in 92.7% of cases. The most frequent recommendations were infusion time (inclusion) (16.7%), dose (appropriateness) (13.0%), dilution/reconstitution (inclusion) (13.0%), and drug withdrawal (13.0%). Regarding the recommendations not accepted, the clinical pharmacist documented the patients were monitored for possible adverse events.

**Table 2:** Problems related to pharmacotherapy according to the pharmaceutical recommendations documented during the pharmacotherapeutic follow-up of patients at the respiratory intensive care unit

Pharmacotherapy-related problems	n (%)	Pharmaceutical recommendations	n (%)
Inadequate timing	3 (1.6)	Timing (appropriateness)	3 (1.6)
Inadequate dilution/reconstitution	15 (8.0)	Purchase of medication/health supplies	2 (1.0)
Dose higher than recommended	24 (12.4)	Correct writing	4 (2.1)
Dose lower than recommended	8 (4.1)	Dilution/reconstitution (appropriateness)	12 (6.3)
Duplicate order/prescription	9 (4.7)	Dilution/reconstitution (inclusion)	25 (13.0)
Unnecessary test	1 (0.5)	Dose (appropriateness)	25 (13.0)
Inadequate pharmaceutical formulation	4 (2.1)	Dose (inclusion)	3 (1.6)
Unavailability (shortage)	19 (9.8)	Pharmaceutical formulation (appropriateness)	2 (1.0)
Missing information in prescription	64 (33.2)	Inclusion of medication	6 (3.1)
Unsafe medication due to drug interaction	10 (5.2)	Technical information on medication	15 (7.8)
Incorrect medication due to contraindication, allergy or adverse reaction	13 (6.7)	Dosage (appropriateness)	6 (3.1)
Necessary medication not administered	1 (0.5)	Dosage (inclusion)	2 (1.0)
Necessary medication not prescribed	6 (3.1)	Medication replaced	23 (12.0)
Unnecessary medication prescribed	4 (2.1)	Cancel unnecessary test orders	1 (0.5)
Adverse drug reaction (ADR)	1 (0.5)	Withdraw medication	25 (13.0)
Incorrect writing	4 (2.1)	Infusion time (appropriateness)	5 (2.6)
No problem in prescription	2 (1.0)	Infusion time (inclusion)	32 (16.7)
Inadequate infusion time	3 (1.6)	Administration route (appropriateness)	1 (0.5)
Inadequate administration route	1 (0.5)		

The problems related to pharmacotherapy (incomplete information in the prescription, inadequate or missing information on pharmaceutical formulation, medication not included on the hospital formulary, among others) were classified regarding severity as potentially lethal (2.1%), severe (14.6%), significant (31.3%), no error (14.6%), and mostly minor (37.5%). Duplicate order, medication omitted from prescription, insufficient dose for the patient's condition, were classified as significant (31.3%). Spelling or interpretation errors that could lead to wrong dispensing, documented drug allergy (such as prescription of dipyrone for patients reporting allergy to this drug), high dose (>10-fold the normal dose of a drug with normal therapeutic index) were classified as severe (14.6%).

Most pharmaceutical recommendations regarding impact were on toxicity, thus reducing the risk of patients on some medications (53.6%) (Table 3).

**Table 3:** Correlation between pharmaceutical recommendations and impact on the study conducted at the respiratory intensive care unit

Pharmaceutical recommendations	Impact		Total %
	Effectiveness %	Toxicity %	
Timing (appropriateness)	-	100 (n=3)	100 (n=3)
Purchase of medication/health supplies	100 (n=2)	-	100 (n=2)
Correction in writing	50 (n=2)	50 (n=2)	100 (n=4)
Dilution/reconstitution (appropriateness)	16.7 (n=2)	83.3 (n=10)	100 (n=12)
Dilution/reconstitution (inclusion)	100 (n=25)	-	100 (n=25)
Dose (appropriateness)	16 (n=4)	84 (n=21)	100 (n=25)
Dose (inclusion)	66.7 (n=2)	33.3 (n=1)	100 (n=3)
Pharmaceutical formulation (adequacy)	100 (n=2)	-	100 (n=2)
Inclusion of medication	100 (n=6)	-	100 (n=6)
Technical information on medication	33.3 (n=5)	66.7 (n=10)	100 (n=15)
Dosage (appropriateness)	33.3 (n=2)	66.7 (n=4)	100 (n=6)
Dosage (inclusion)	100 (n=2)	-	100 (n=2)
Medication replaced	78.3 (n=18)	21.7 (n=5)	100 (n=23)
Cancel unnecessary test orders	-	100 (n=1)	100 (n=1)
Medication withdrawal	8 (n=2)	92 (n=23)	100 (n=25)
Infusion time (appropriateness)	60 (n=3)	40 (n=2)	100 (n=5)
Infusion time (inclusion)	37.5 (n=12)	62.5 (n=20)	100 (n=32)
Administration route (appropriateness)	-	100 (n=1)	100 (n=1)

n: represents the number of pharmaceutical recommendations given and classified according to impact.

The drug class more often involved in pharmacotherapy-related problems related was antimicrobials, in general, for systemic use (53%). The pharmaceutical recommendations for these agents had an impact on effectiveness and toxicity of pharmacotherapy. Drugs for digestive system and metabolism (14%) rank second. In these groups, meropenem, piperacillin/tazobactam and omeprazole stood out (Table 4).

**Table 4:** Correlation between classification of the drugs involved according to the Anatomical Therapeutic Chemical classification system and impact of pharmacotherapy-related problems

ATC Classification		Impact		Total %
		Effectiveness %	Toxicity %	
A	Digestive system and metabolism	67.9 (n=19)	32.1 (n=9)	100 (n=28)
B	Blood and hematopoietic organs	7.7 (n=1)	92.3 (n=12)	100 (n=13)
C	Cardiovascular system	23.5 (n=4)	76.5 (n=13)	100 (n=17)
D	Dermatological medications	100 (n=1)	-	100 (n=1)
H	Systemic hormone preparations, excluding sex hormones and insulin	33.3 (n=3)	66.7 (n=6)	100 (n=9)
J	Antimicrobials in general for systemic use	51.9 (n=55)	48.1 (n=51)	100 (n=106)
M	Musculoskeletal system	50 (n=1)	50 (n=1)	100 (n=2)
N	Nervous system	15 (n=3)	85 (n=17)	100 (n=20)
P	Antiparasitary, insect repellents	100 (n=2)	-	100 (n=2)
R	Respiratory system	-	100 (n=2)	100 (n=2)

χ^2^ test: p<0.05. n: represents the number of medications implied in the pharmacotherapy-related problems and classified according to impact. ATC: Anatomical Therapeutic Chemical.

## DISCUSSION

The multiprofessional residency program allowed the inclusion of pharmacists in the wards, ICU, and outpatient clinics of the hospital, as well as implementation of clinical activities. Pharmacotherapeutic follow-up of patients in the HM respiratory ICU is done by monitoring medications used and duration of use, including antimicrobials and treatment of comorbidities, to identify problems related to medications, prevent and/or solve them, with a focus on patient safety. The form employed has a field to document pharmaceutical recommendations and results of laboratory tests used for monitoring.

In a study conducted by Bohomol et al.,^(^ ^20^ ^)^ the mean age was 58 years and the mean length of stay 12.4 days. However, a study carried out in a Dutch hospital found different data; there was predominance of male patients, with a mean age of 63.22 years and an mean length of stay of 2.06 days.^(^ ^1^ ^)^ These data corroborate our findings. A prospective cohort study in Japan had mostly male patients, with mean age of 66 years and mean length of stay of 10 days.^(^ ^4^ ^)^ The main issue faced when studying an older population, with more than one diagnosis and hospitalized for many days at an ICU, is the increased risk of adverse events. These are defined as unwanted complications arising from care provided, not attributed to the natural course of the underlying disease, caused mainly by problems related to prescription.^(^ ^21^ ^)^

The participation of the pharmacist in the daily clinical activities of inpatients units was a major advance at the HM during the study period, and enabled identifying the problems related to pharmacotherapy that were not perceived at pharmacy, such as drug interactions, incompatibilities, timing, dilution, inadequate doses, among others. All prescriptions made during the patients’ stay were evaluated and validated, *i.e*., one prescription per day for each patient. Based on the problems found, the pharmacist made pharmaceutical recommendations to prevent them from harming patients.

The benefit of having a pharmacist involved in clinical activities can be confirmed by the number of pharmacotherapy-related problems identified in the prescriptions analyzed in the study. This result is similar to that found by Agalu et al., who reported a 23.8% rate of missing information in the prescriptions (dose, frequency, route of administration, and unit of measurement), and 15.1% related to dose errors.^(^ ^22^ ^)^ Klopotowska et al., also demonstrated that most problems related to pharmacotherapy were linked to dose errors or omission (31.6%).^(^ ^1^ ^)^

In this study, the importance of individualizing pharmacotherapy was demonstrated by the clinical pharmacist through the most frequent recommendations. Studies conducted at university hospitals in Fortaleza (CE, Brazil), Curitiba (PR, Brazil), and in the Netherlands, also detected the need for management of dilution and infusion time, dose adjustment and drug withdrawal.^(^ ^1^ ^,^ ^5^ ^,^ ^23^ ^)^ Costa, in a study carried out at a university hospital in Campinas (SP, Brazil), showed acceptance of 86.14% of pharmaceutical recommendations made in one year.^(^ ^8^ ^)^

Leape et al., however, in a study conducted in the United States, had 99% acceptance.^(^ ^13^ ^)^

The drug classes more often involved in pharmacotherapy-related problems study were antimicrobials, in general, for systemic use, and agents for the digestive system and metabolism. Such drugs are usually prescribed for critical patients, for being part of clinical protocols (*e.g*., omeprazole for prophylaxis of stress ulcer) or because they are used to treat common diseases in this population (such as meropenem, for Gram-negative bacteria infections). Other studies have also shown these drugs to be the most frequently responsible for pharmacotherapy-related problems.^(^ ^3^ ^,^ ^5^ ^,^ ^8^ ^,^ ^23^ ^)^

As limitation of this study, we can mention a flaw in the documentation of pharmaceutical results for further analysis, as well as records of problems for analysis of severity.

## CONCLUSION

Patients admitted to the respiratory intensive care unit, where pharmacotherapeutic follow-up was assessed, were on polypharmacy for presenting more than one diagnosis. Therefore, potentially lethal and severe problems were detected, and pharmaceutical recommendations were made to the multiprofessional team. The recommendations led to reduced toxicity and increased effectiveness of drug therapy prescribed.
